# *OsDGD2β* is the Sole Digalactosyldiacylglycerol Synthase Gene Highly Expressed in Anther, and its Mutation Confers Male Sterility in Rice

**DOI:** 10.1186/s12284-019-0320-z

**Published:** 2019-08-14

**Authors:** Rasbin Basnet, Nazim Hussain, Qingyao Shu

**Affiliations:** 1 National Key Laboratory of Rice Biology, Institute of Crop Sciences, Zhejiang University, Hangzhou, Zhejiang China; 2 Hubei Collaborative Innovation Center for the Grain Industry, Yangtze University, Jingzhou, 434025 Hubei China; 3 Zhejiang Key Laboratory of Crop Germplasm Resources, Institute of Crop Sciences, Zhejiang University, Hangzhou, Zhejiang China

**Keywords:** Rice, OsDGD2β, DGDG, Digalactosyldiacylglycerol, Male sterility, Galactolipids

## Abstract

**Background:**

Digalactosyldiacylglycerol (DGDG) is one of the major lipids found predominantly in the photosynthetic membrane of cyanobacteria, eukaryotic algae and higher plants. DGDG, along with MGDG (Monogalactosyldiacylglycerol), forms the matrix in thylakoid membrane of chloroplast, providing the site for photochemical and electron transport reactions of oxygenic photosynthesis.

**Results:**

In silico analysis reveals that rice (*Oryza sativa* L.) genome has 5 genes encoding DGDG synthase, which are differentially expressed in different tissues, and *OsDGD2β* was identified to be the sole DGDG synthase gene expressed in anther. We then developed *osdgd2β* mutants by using the CRISPR/Cas9 system and elucidate its role, especially in the development of anther and pollen. The loss of function of OsDGD2β resulted in male sterility in rice characterized by pale yellow and shrunken anther, devoid of starch granules in pollen, and delayed degeneration of tapetal cells. The total fatty acid and DGDG content in the anther was reduced by 18.66% and 22.72% in *osdgd2β*, affirming the importance of DGDG in the development of anther. The mutants had no notable differences in the vegetative phenotype, as corroborated by relative gene expression of DGDG synthase genes in leaves, chlorophyll measurements, and analysis of photosynthetic parameters, implying the specificity of OsDGD2β in anther.

**Conclusion:**

Overall, we showed the importance of DGDG in pollen development and loss of function of OsDGD2β results in male sterility. Here, we have also proposed the use of *OsDGD2β* in hybrid rice breeding using the nuclear male sterility system.

**Electronic supplementary material:**

The online version of this article (10.1186/s12284-019-0320-z) contains supplementary material, which is available to authorized users.

## Background

Lipids are essential components in all living cells, comprising ~ 5 to 10% by dry weight and possessing diverse functions in protection, cellular metabolism and carbon storage (Ohlrogge and Browse 1995; Cassim et al. 2019). In animals, phospholipids are the most abundant membrane lipids, while in plant, galactolipids are the predominant, constituting ~ 75% of total membrane lipids in leaves (Dörmann and Benning 2002; Nakamura 2017). The galactolipids in the photosynthetic membranes of higher plants mostly consist of monogalactosyldiacylglycerol (MGDG) and digalactosyldiacylglycerol (DGDG), accounting for 50% and 20% of the chloroplast lipid, respectively (Dörmann and Benning 2002; Kalisch et al. 2016). In chloroplast, these galactolipids provide the lipid matrix for the thylakoid membrane, which is the site of the photochemical and electron transport reactions (Mizusawa and Wada 2012; Dörmann 2013; Kobayashi and Wada 2016). The composition of lipid is highly unique and conserved in the thylakoid membrane among oxygenic photosynthetic organisms, where they are involved not only in the formation of the lipid bilayers, but also in the folding and assembly of the protein subunits in photosynthetic complexes (Sakurai et al. 2007; Boudière et al. 2014; Kobayashi et al. 2016). MGDG and DGDG have also been reported to be found in various other organelles including tonoplast, endoplasmic reticulum, and golgi membranes, indicating the importance of these galactolipids in non-photosynthetic processes (Hartel et al. 2000; Wang et al. 2014).

In plants, galactolipid synthesis occurs by adding a galactose from UDP-galactose onto the diacylglycerol (DAG) backbone to form MGDG by the enzyme MGDG synthase. Similarly, DGDG is synthesized from MGDG by transferring a second galactose onto the MGDG by the enzyme DGDG synthase (Ohlrogge and Browse 1995; Mizusawa and Wada 2012). DGDG synthesis in *Arabidopsis* is encoded by two genes, *AtDGD1* and *AtDGD2* (Kelly et al. 2003). AtDGD1 has been found to be the principal contributor of DGDG in chloroplast and the *atdgd1* mutants had more than 90% reduction in the DGDG content (Dörmann et al. 1995) and showed stunted growth, pale green leaf colour, reduced photosynthetic capability, and decreased PSII/PSI ratio (Hartel et al. 1997; Dörmann et al. 1999; Boudière et al. 2014; Lin et al. 2016). The double mutant, *atdgd1* and *atdgd2* was found to have more severe phenotype compared to *atdgd1* (Kelly et al. 2003), while the *atdgd2* mutants didn’t show any alteration in the growth and development, with no differences in the chlorophyll content, suggesting that AtDGD2 is not essential in polar lipid synthesis under optimal growth conditions (Kelly et al. 2003; Mizusawa and Wada 2012). However, under the phosphate starvation conditions, AtDGD2 has been reported to be responsible for synthesizing the residual DGDG (Kelly and Dörmann 2002; Jouhet et al. 2004), which is exported to various extra-plastidial membranes, substituting for phospholipids to maintain cell membrane homeostasis (Hartel et al. 2000; Boudière et al. 2014).

**Fig. 1:**
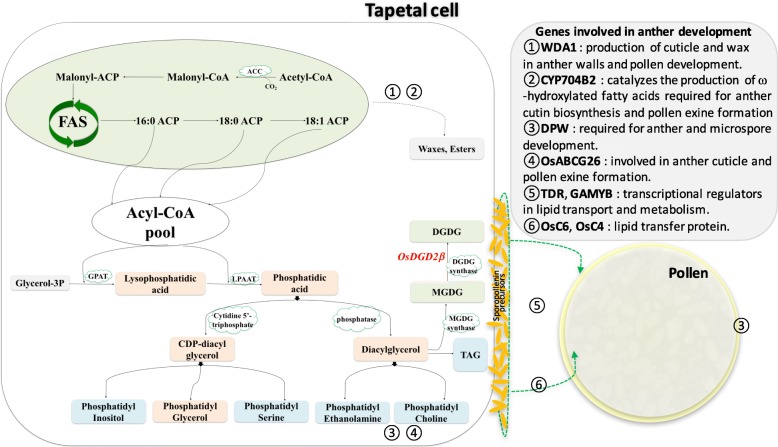
Simplified diagram showing biosynthesis of lipids involved in anther development in rice. De-novo synthesis of fatty acid occurs in plastid (shaded green) while the pathways for synthesis of Phosphatidic acid, CDP-Diacylglycerol and Diacylglycerol are present in both plastid and endoplasmic reticulum (ER) (shaded orange). MGDG and DGDG are synthesized in the plastid; TAG and other Phospholipids (Phosphatidylcholine, Phosphatidylethanolamine, Phosphatidylserine, Phosphatidylinositol) are synthesized in ER (shaded blue); while Phosphatidylglycerol is synthesized in plastid as well as ER and mitochondria. The circled numbers represent the most active site of the corresponding protein localization or function. ACC, acetyl-CoA carboxylase; ACT acetyl-CoA transferase; ACP, Acyl carrier protein; LPAT, Lysophosphatidic acid transferase; MGDG, Monogalactosyldiacylglycerol; DGDG, Digalactosyldiacylglycerol; TAG, Triacylglycerol

Though DGDG has been investigated thoroughly in the photosynthetic tissues of many photosynthetic organisms, research on their importance in the development of flower or seed are still limited. The galactolipid synthesizing genes have been reported to be expressed in floral tissues of *Arabidopsis, Petunia*, and *Lilium* (Nakamura et al. 2003, 2009; Kobayashi et al. 2004; Nakamura 2015), and substantial amount of galactolipids have been quantified in pollen of rapeseed (Evans et al. 1990). In anther and pollen, lipids are found to be essential constituents, where they not only provide protection to the male gametes, but also function in pollen-stigma communication (Edlund et al. 2004; Ariizumi and Toriyama 2011). Lipids in anther are synthesized in the tapetum and several genes have already been identified in rice including *WDA1* (Jung et al. 2006), *DPW* (Shi et al. 2011), *CYP704B2* (Li et al. 2010), *CYP703A3* (Yang et al. 2014), *OsGPAT3* (Men et al. 2017). Lipids originated in the tapetal cells are then transported to the developing microspore by lipid transfer proteins (LTPs) such as those encoded by *OsC6* and *OsC4* (Zhang et al. 2010, 2016). Various regulatory genes such as *TDR* (Zhang et al. 2008), *GAMYB* (Kaneko et al. 2004; Shi et al. 2015) play a key role in controlling the expression of various genes involved in anther development (Fig. 1). Tapetal lipids are essential for the morphology and functionality of anther and pollen, and loss of function of any of these lipid biosynthesizing genes or their regulatory factors was found to result abnormality in pollen development or even male sterility (Ariizumi et al. 2004; Ariizumi and Toriyama 2011; Zhang et al. 2016).

Even though the cruciality of lipids in anther development has been evident, the various classes of lipids present in anther has not been classified and studied in-depth yet. In this report, we noticed DGDG as one of the major lipids in anther and identified five genes encoding DGDG in rice. We also studied their genomic structures, conserved motifs and domain, and phylogenetic relationship with the *Arabidopsis* DGDG synthase genes. *OsDGD2β* was found to be highly expressed in anther, and we employed the CRISPR/Cas9 system to generate mutants of *OsDGD2β* and investigated its role in the development of anther and pollen.

## Results

### Identification and characterization of DGDG synthase genes in rice

#### DGDG synthase in rice is encoded by five genes

Five genes encoding DGDG synthase were identified in rice genome through homologous search of *Arabidopsis* DGDG synthase genes (Additional file 1: Table S8). The gene information along with peptide length, molecular weight and isoelectric points (PIs) are also listed in Additional file 1: Table S8. Gene names were designated on the basis of their phylogenetic relationship with their orthologues in *Arabidopsis*. Three genes orthologous to *AtDGD1* were designated as *OsDGD1α, OsDGD1β* and *OsDGD1δ,* while the two orthologous to *AtDGD2* were assigned name as *OsDGD2α* and *OsDGD2β* (Additional file 2: Figure S1A). The gene structures showed that the sequences are not well conserved among the rice DGDG synthase genes. *OsDGD1α*, *OsDGD1β* and *OsDGD1δ* have 7 exons similar to *AtDGD1*, while *OsDGD2α* and *OsDGD2β* have only 4 exons, which is similar to *AtDGD2* (Additional file 2: Figure S1B). *OsDGD1α*, *OsDGD1β*, *OsDGD1δ,* and *OsDGD2α* have single transcript, while *OsDGD2β* has two transcripts. These two transcripts of *OsDGD2β* differ in their gene structure and protein length, but share the same third and fourth (last) exon.

There are 10 motifs conserved between *Arabidopsis* and rice DGDG synthases (Additional file 2: Figure S1C), which are mostly located towards the C-terminal end. All these DGDG synthases contain a single common domain Glycos_transf_1 (PF00534), which is associated with the motifs 1, 6 and 9 (Additional file 2: Figure S1C), and spanned between two last exons of DGDG synthase in both rice and *Arabidopsis*. Therefore, the functional region of the DGDG synthase could be towards the mid and C-terminal end, where they share common motifs and a domain.

#### *OsDGD2β is* expressed highly in anther

The RNA-seq FPKM expression values of rice DGDG synthesis genes showed the expression of *OsDGD1α* was the highest in leaf, followed by *OsDGD1δ* and *OsDGD2β* (Additional file 3: Figure S2A). While in the anther, *OsDGD2β* was found to be the only DGDG synthesis gene with an exceptionally high expression. The expression of *OsDGD2β* was also noticed the highest in pistil, endosperm and embryo, while the expression of other DGDG synthesis genes in floral tissues and seeds were very low or negligible. The expression pattern of DGDG synthesis genes in *Arabidopsis* and maize was not found conserved with those in rice (Additional file 3: Figure S2 B&C). In *Arabidopsis*, *AtDGD1* was found highly expressed than *AtDGD2* in all tissues. However, in maize, the expression of *ZmDGD1δ* was found highest in the leaf while *ZmDGD2β* was highest in the anther which is similar to its monocot counterpart, rice. (Additional file 3: Figure S2C).

Based on transcriptome data (Deveshwar et al. 2011), *OsDGD2β* and *OsDGD1β* were the only DGDG synthesis genes with detectable expression. The expression level of *OsDGD2β* was ~ 113, ~ 193, ~ 225 and ~ 124 times higher than that of *OsDGD1β* anthers at PMA (pre-meiotic anther), MA (meiotic anther), SCP (anther with single celled pollen) and TPA (tri-nucleate pollen) stages, respectively (Additional file 3: Figure S2D). In addition, the expression of both *OsDGD2β* and *OsDGD1β* were substantially elevated by 3.4 and 4 fold, respectively, at the TPA stage while it remained relatively stable at other stages.

#### OsDGD2β is localized in chloroplast

**Fig. 2:**
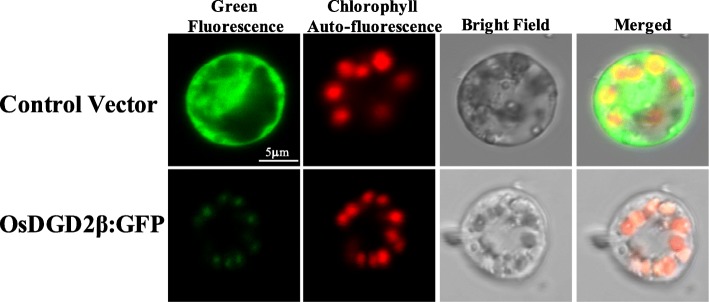
Subcellular localization of OsDGD2β via transient expression of 35S:OsDGD2β:GFP in rice protoplast. Control vector (35S:GFP) has bright GFP signal distributed throughout the cell. 35S:OsDGD2β:GFP has fluorescent signal localized in the chloroplast, confirmed by the red auto-fluorescent signal from chlorophyll

Green fluorescent signal was observed from the GFP (Green fluorescence protein) fused proteins transiently expressed in the protoplast of rice. The chloroplasts were visualized by the auto fluorescence of chlorophyll and appear at the periphery of the protoplast in red. The control vector (35S:GFP) has bright GFP signal distributed throughout the cell, while the fluorescence from 35S:OsDGD2β:GFP was localized only in the chloroplast (Fig. 2), affirming the localization of OsDGD2β in the plastid.

### Generation of *osdgd2β* mutants and their characteristics

#### Two *osdgd2β* mutant lines were generated

**Fig. 3:**
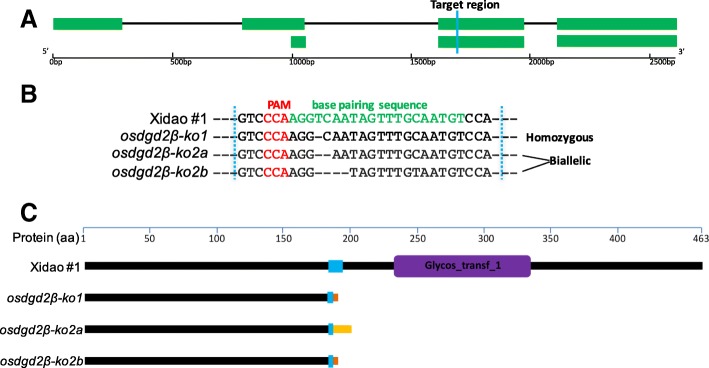
Targeted mutagenesis of *OsDGD2β* using CRISPR/Cas9 system. **a** Two transcripts of *OsDGD2β* with the target region shown in blue. **b** DNA sequences of the target region with PAM and genome specific sgRNA sequence shown in red and green, respectively. *osdgd2β-1* is a homozygous mutant with 1-bp deletion while *osdgd2β-2* is a biallelic mutant with 2-bp (*osdgd2β-2a)* and 5-bp (*osdgd2β-2b)* deletion. **c** schematic representation of truncation in protein translation in the mutants. Purple colour depicts the only domain (Glycos_transf_1), changes in amino acid sequences in the mutants’ protein are shown in brown and yellow colour after the target region

In T_0_ plants, three types of mutations were found. One homozygous T_0_ plant had a 1-bp deletion while another plant with bi-allelic mutations had 2-bp and 5-bp deletions. The mutation sites in the target region were all 3-bp away from the PAM region (Fig. 3a and b). The two mutant lines were designated as *osdgd2β-1* and *osdgd2β-2*. All the mutants had protein translation terminated immediately after the mutation point before the Glyco-transf_1 domain (Fig. 3c), suggesting all three mutations are knock-out mutations.

#### Changes in DGDG and total fatty acids content in mutant plants

**Fig. 4:**
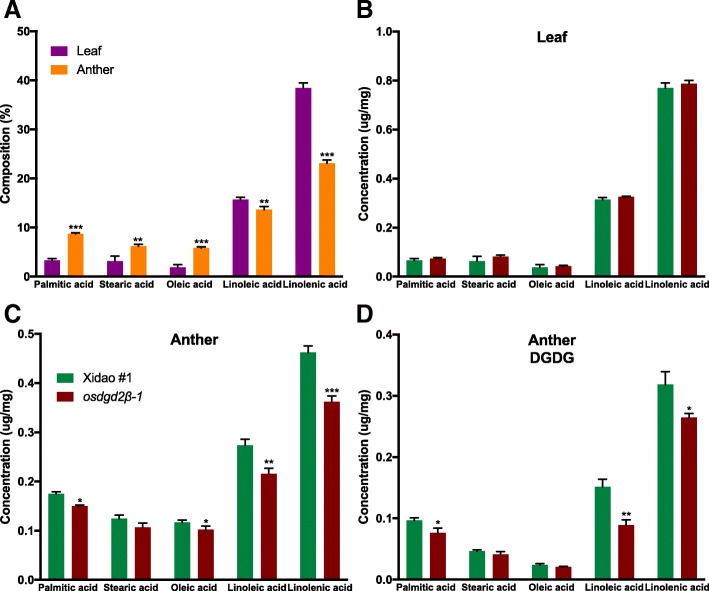
Fatty acid composition in leaf and anther of wild-type cultivar Xidao #1 and its mutant *osdgd2β-1****.*** **a** shows the fatty acid composition in leaf (purple) and anther (orange) in weight percentage of wild-type. **b** & **c** show concentration of fatty acids in leaf and anther, respectively. **d** shows concentration of fatty acids in DGDG spot obtained from TLC (refer Additional file 4: Figure S3)*.* All values represent means ± standard deviations. Asterisk represent statistically significant differences (Tukey’s test, * *P* < 0.05, ** *P* < 0.01, *** *P* < 0.001). Fatty acid concentration in **b**, **c** and **d** are measured on the basis of fresh weight of tissue samples

GC-FID (Gas chromatography flame ionization detector) analysis revealed that the principal fatty acid in both leaf and anther was linolenic acid (18:3) followed by linoleic acid (18:2) (Fig. 4). Palmitic acid (16:0), stearic acid (18:0) and oleic acid (18:1) were also detected in considerable amount, whereas other fatty acids were negligible (and not included in this study). The percentage of linolenic acid and linoleic acid was found higher in leaf than in anther, whereas palmitic acid, stearic acid and oleic acid were higher in anther than in leaf (Fig. 4a). There was no significant difference in the fatty acid composition in leaves between wild-type and mutants (Fig. 4b). However, in the anther, the total fatty acid content was reduced significantly by 18.66%. Similarly, all the individual fatty acids were also reduced significantly except stearic acid (Fig. 4c). The reduction in linolenic acid was found highest (21.6%), followed by linoleic acid (21.15%), palmitic acid (14.17%) and oleic acid (14.12%).

The lipid spots separated on the TLC (Thin layer chromatography) plates showed DGDG is the principal lipid class in the anther (the MGDG spots were very small), and no visual differences were noted between wild-type and its mutants (Additional file 4: Figure S3). DGDG spot trans-esterified and analysed by GC-FID showed that DGDG comprises about 55% of the total lipid content in the anther in the wild-type (Fig. 4c and d). In the mutants, fatty acid methyl esters (FAMEs) content of DGDG was significantly reduced by 22.72%, with the highest reduction in linoleic acid (41.10%) followed by palmitic acid (20.91%) and linolenic acid (16.90%), while no significant reduction was noticed in stearic acid and oleic acid in the mutant (Fig. 4d).

#### The *osdgd2β* mutants are male sterile

No visual differences in the vegetative development was noticed between the wild-type and *osdgd2β* mutants. The chlorophyll content and photosynthetic parameters were also not significantly different (Additional file 5 Figure S4 & Additional file 6: Figure S5). However, at the flowering stage, the exertion of panicles from the leaf sheath were not complete in the mutants, as compared to the wild-types. Unlike in wild-type, anthers in most of the spikelets in mutants didn’t not exert out at the time of flowering (Additional file 7: Figure S6).

**Fig. 5:**
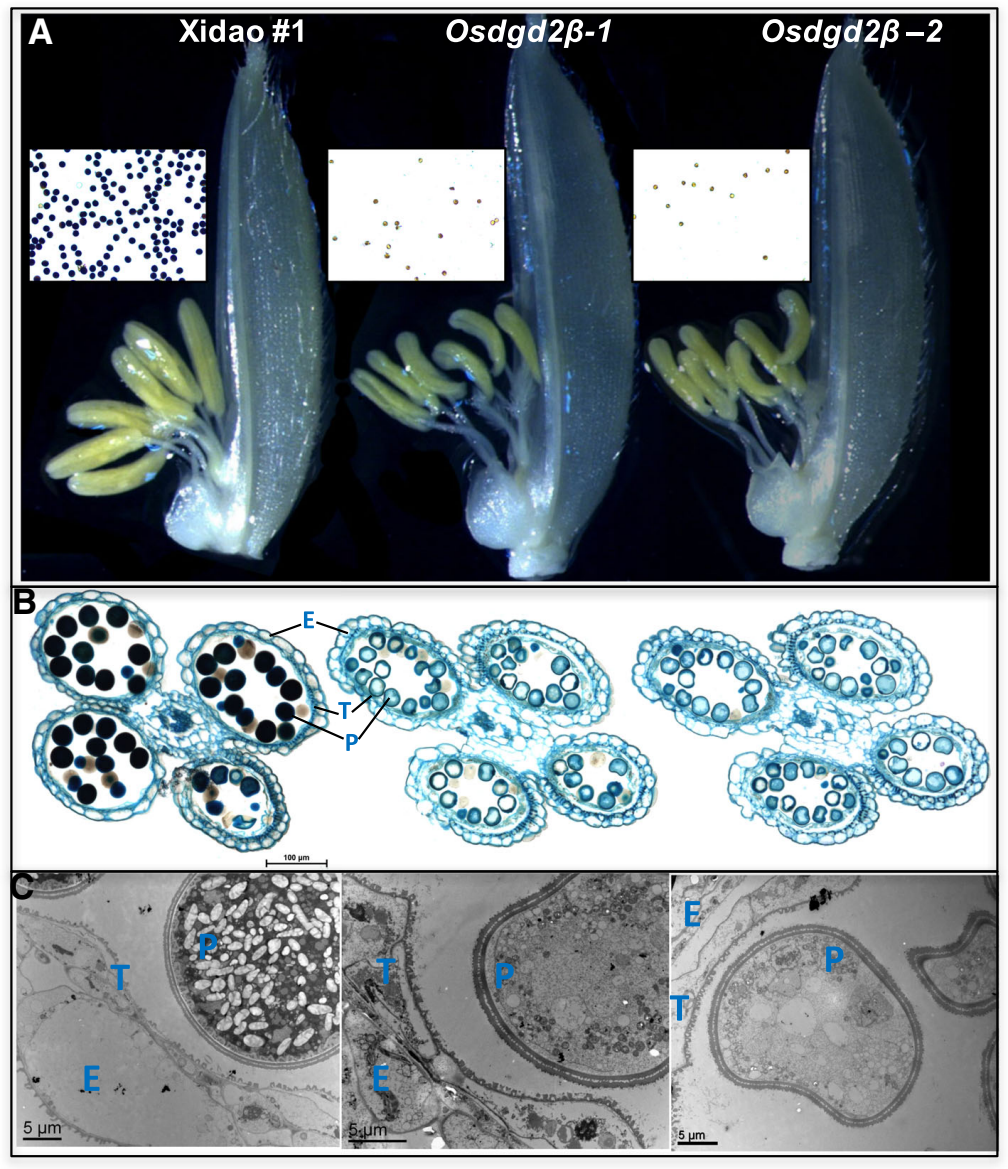
Phenotypic observation of anther of wild-type cultivar Xidao #1 and its mutants *osdgd2β-1 and osdgd2β-2*. **a** spikelet with palea removed showing differences in anther with pollen stained with 1% IKI (iodine potassium iodide) in the middle, **b** Microscopy of cross section of anthers stained with 0.5% toluidine blue, and **c** Transmission Electron Microscopy (TEM) @4000X magnification. E epidermis, T tapetum, P pollen

The anthers in *osdgd2β* were comparatively smaller, shrunk and curved (Fig. 5a). The anthers in wild-types were bright-yellow with pollens round in shape and deeply stained by iodine, while anthers in *osdgd2β* were faint yellow with fewer and unstained pollens (Fig. 5b). These results suggest male sterility, which is further corroborated by the failure of mutants to set any seeds on their own. To assess whether the mutations also affect female fertility, the mutants were emasculated and cross pollinated with pollen from wild-type. Normal seed set was observed in those panicles (Additional file 7: Figure S6C), suggesting that the mutants are female fertile.

Microscopic examination also showed the mutant anthers had unstained pollens, smaller in size and shrunk as compared to wild-types (Fig. 5a). In the wild-type, the tapetal layer was almost completely degenerated, whereas, in the mutants the tapetal layer persisted as a thick single layer tissue (Fig. 5b&c). The pollen grains under TEM (Transmission electron microscopy) showed wild-type pollens loaded with starch granules and other components, whereas mutant pollens were completely devoid of any starch granules (Fig. 5c).

### Effect of *OsDGD2β* mutation on transcription of genes involved in lipid biosynthesis and transport in anther

**Fig. 6:**
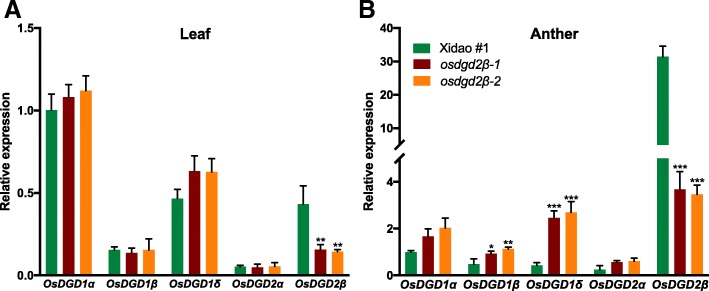
Relative gene expression levels of DGDG synthesis genes in leaf (**a**) and anther (**b**) of a wild-type cultivar Xidao #1 and its mutants *osdgd2β-1* and *osdgd2β-2*. The expression levels were first normalized to the internal control gene *OsACTIN* and reported relative to the expression level of *OsDGD2β* in wild-type (assigned a value of 1). All values represent means ± standard deviations. Asterisk represent statistically significant differences (Tukey’s test, * *P* < 0.05, ** *P* < 0.01, *** *P* < 0.001)

**Fig. 7:**
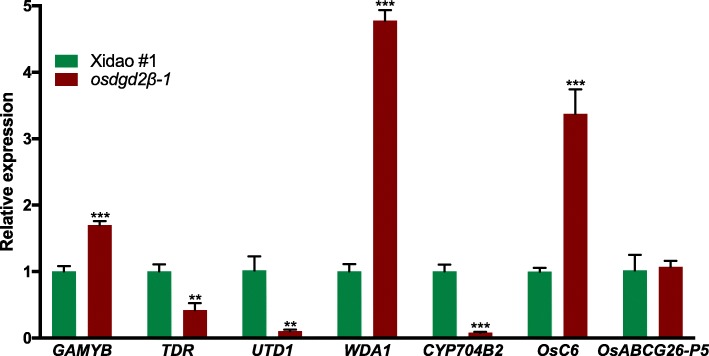
Relative expression level of genes involved in anther development of a wild-type cultivar Xidao #1 and its mutant *osdgd2β-1****.*** The expression levels were normalized to the internal control gene *OsACTIN*. All values represent means ± standard deviations. Asterisk represent statistically significant differences (Tukey’s test, * *P* < 0.05, ** *P* < 0.01, *** *P* < 0.001)

The expression of the mutated gene, *OsDGD2β,* was significantly decreased in both leaf (~ 65.26%) and anther (~ 88.62%) (Fig. 6) in the mutants. No significant changes in the expression of other DGDG synthase genes was observed in leaf (Fig. 6a), while in the anther the differences were significant except that of *OsDGD2α* (Fig. 6b)*. OsDGD1δ*, *OsDGD1β* and *OsDGD1α* had ~ 5.9, ~ 2.1 and ~ 1.8 fold increase in their expression in the mutant anther, respectively. The deleterious mutations in *osdgd2β* also affected the expression of some of the other genes involved in anther development (Fig. 7). We found a significant increase in the expression of *WDA1* (3.8 fold) along with *OsC6* (2.4 fold) and *GAMYB* (0.8 fold) in the mutant anthers. On the other hand, the expression of *TDR* was found reduced (0.6 fold), while expression of *OsC4* was found negligible at the flowering stage in the mutant anther.

## Discussion

### *OsDGD2β* is the sole highly expressed DGDG synthase gene in anther

Five genes were identified in rice for the synthesis of DGDG, sharing similarity with those in *Arabidopsis* in terms of gene structure, motifs and domain, indicating the common structural and functional characteristics. The subcellular localization of OsDGD2β was also similar to both AtDGD1 and AtDGD2 (Kelly et al. 2003), suggesting plastid specific function of OsDGD2β. In *Arabidopsis*, AtDGD1 is responsible for synthesizing the bulk of DGDG for thylakoid membrane biogenesis, while the expression of AtDGD2 is mainly increased during the phosphate deprivation condition (Kelly and Dörmann 2002; Jouhet et al. 2004). Our comparative gene expression analysis also shows *AtDGD1* and its orthologues *OsDGD1α* in rice, and *ZmDGD1α* and *ZmDGD1δ* in maize have the highest expression in leaf. However, unlike in *Arabidopsis*, the expression of *OsDGD2β* was found the highest in anther along with pistil, embryo and endosperm. The expression pattern of maize DGDG synthesis genes also showed similarity to that of rice, but unlike in rice, there is no gene expressed solely in anther and the expression of *ZmDGD* genes in anther are lower than in leaf. These suggest that expression of DGDG synthase genes are not well conserved among rice, maize and *Arabidopsis*.

The high expression of *OsDGD2β* in all stages of anther development also indicates its importance in DGDG synthesis in rice anther. The elevated expression of both *OsDGD2β* and *OsDGD1β* at TPA stage further suggests DGDG become more important in later stage of pollen development, which is characterized by mitotic division of microspore, complete degeneration of tapetum and accumulation of starch and lipidic materials in the pollen grain (Zhang et al. 2011). Hence, the anther specific expression of *OsDGD2β*, particularly at the TPA stage, provides strong evidence for the anther-specific function of DGDG encoded by *OsDGD2β*.

In this study, *OsDGD2β* was shown not to have any contribution in the development of vegetative parts since there was not any significant differences in the chlorophyll content, photosynthesis rate, stomatal conductance and transpiration rate between wild-type and mutants. Moreover, there was no change in the expression of other DGDG synthase genes in the *osdgd2β* leaf to compensate for the mutation of *OsDGD2β.* These results are also in accordance with its orthologous in *Arabidopsis*, where the *atdgd2* mutant didn’t show any marked phenotypic differences under normal conditions, however the double mutant of *atdgd1* and *atdgd2* showed more stunned growth than single mutant of *atdgd1* (Dörmann et al. 1995; Kelly et al. 2003). The lipid analysis in leaf also entails similar result with no significant changes in the fatty acid composition between wild-type and the mutants, suggesting the lack of contribution of OsDGD2β to synthesize DGDG required for photosynthesis. However, in the anther, the mutation of *OsDGD2β* affected the expression of other genes as well as decreased the fatty acid content, confirming the specificity and essentiality of *OsDGD2β* in anther development.

### Mutation of *OsDGD2β* generates male sterile rice

The morphology of pollen wall varies among different species, and is basically comprised of three layers: the pollen coat, the outer exine layer and the inner intine layer. These layers are mainly composed of lipids and their derivatives such as fatty acids, waxes, and phospholipids, and disruption of pollen wall structure leads to pollen degradation and/or abortion, leading to partial or complete male sterility (Wilson and Zhang 2009; Shi et al. 2015). The loss of function of OsDGD2β resulted in male sterility in rice, however, its orthologue *atdgd2* did not have any significant phenotypic changes in *Arabidopsis*. This could be due to the differences in the mechanism and pathways for the development of anther and pollen between monocots and dicots (Shi et al. 2015). The pollen grains of rice have smooth surface with particulate exine patterning and more inter-layer space, while in *Arabidopsis,* pollen grains have ellipsoidal epidermis covered with elegant reticulate cavities (Li and Zhang 2010; Zhang et al. 2011, 2016). In *Arabidopsis*, exine has thin nexine layer, semi open tectum layer and longer baculum, with an abundant pollen coat deposited in the sculptured cavities in exine, while in rice, tectum and nexine are thicker, and higher density of bacula, with much less pollen coat filled in the spaces between tectum and nexine (Ariizumi and Toriyama 2011; Zhang et al. 2016). The spaces between the sexine and nexine also determines the species-specific structure of the pollen grains, rice having more inter-layer spaces compared to *Arabidopsis* (Li and Zhang 2010; Shi et al. 2015). These evidences signify the diversification of the development of anther and pollen between rice and *Arabidopsis* during evolution.

Several genes in rice have been reported to be involved in the synthesis and secretion of tapetal lipids such as Wax-deficient anther 1 (*WDA1*), Defective pollen wall (*DPW*), *CYP70B2* and *Fax1*, and are vital for pollen fertility (Jung et al. 2006; Zhang et al. 2016). Similarly, mutations in other rice sporopollenin biosynthesis genes such as *DPW*, *CYP704B2*, and *CYP703A3* have been found to cause defective sporopollenin biosynthesis with defective anther cuticle (Zhang et al. 2010; Shi et al. 2015). Likewise, *OsC6*, which encodes a LTP, is essential for post meiotic anther development in rice, particularly in the pollen exine development (Zhang et al. 2010). Some of the key regulators controlling the expression of these genes are *TDR* (Tapetum Degeneration Retardation) and *GAMYB* (Shi et al. 2015). *TDR*, mainly expressed in tapetum, is essential for the degradation of tapetum, and the mutant of this gene result in complete male sterility in rice (Zhang et al. 2008). *WDA1* is involved in synthesis of very long chain fatty acids, and the loss of function of *WDA1* results in abnormal development of anther epicuticular wax crystals with defective pollen exine formation (Jung et al. 2006). Some of these functional and regulatory genes showed remarkable variations in their expressions in *osdgd2β* anther. The increased *WDA1* transcripts suggest the loss of function of OsDGD2β might have diverged the fatty acid pool towards fatty acid elongation pathway in synthesizing longer fatty acids chains such as waxes, thereby increasing the level of *WDA1* transcription. This might have consequently increased the expression of a LTP, *OsC6* and its transcriptional regulator *GAMYB*. The expression of *OsABCG26*, which is required for anther cuticle and pollen exine formation, was found unchanged in the *osdgd2β* mutants (Chang et al. 2016b). On the other hand, the expression of *TDR* was found significantly decreased, and therefore have lesser degeneration of tapetum in the *osdgd2β* as observed in this study. Another tapetal specific gene *CYP704B2* also has reduced expression in *osdgd2β* and share some similarities with *cyp704B2* mutant such as abortive pollen and swollen sporophytic tapetal layer (Li et al. 2010). Hence, lipids and their derivatives are crucial for the development of anther and fertility of the pollens, and disruption of any of these lipids synthesizing genes leads to pollen degradation and/or abortion, leading to partial or complete male sterility (Shi et al. 2015).

Lipids in the anther are originated in the tapetum (Piffanelli et al. 1998), which is the innermost layer of the anther. Tapetum supports the development of microspores by providing enzymes, nutrients, metabolites, in the form of sporopollenin precursors (Piffanelli et al. 1998; Zhang et al. 2008, 2016). In many cereals such as rice and wheat, tapetum also produces particles called Ubish bodies, which transport sporopollenin precursors to the microspore to synthesize exine (Wang et al. 2003; Ariizumi and Toriyama 2011). However, such Ubish bodies have not been identified in *Arabidopsis* and other *Brassicaceae*. The tapetum undergoes degradation by programmed cell death (PCD) after the release of microspores from tetrad, and the proteins and lipids in the sporopollenin precursors are deposited on the pollen, particularly in the exine (Piffanelli et al. 1998; Edlund et al. 2004; Zhang et al. 2016). The lipid in the exine confers hydrophobicity and provides resistant to extreme physical and chemical changes such as high temperature, desiccation, ultraviolet (UV) irradiation and mechanical damages (Dickinson et al. 2000; Ariizumi and Toriyama 2011; Zhang et al. 2016). Lipids in exine has an important role in shape and patterning of the pollen and is of paramount importance for the pollen stigma interactions (Dickinson et al. 2000; Wheeler et al. 2001; Edlund et al. 2004).

DGDG, in particular, has been found critical for bilayer formation in the membranes conferring membrane stability and providing tolerance to high temperature stresses (Chen et al. 2006; Kalisch et al. 2016). The high proportion of DGDG in anther noticed in this study indicates DGDG as one of the key lipids important for biogenesis and viability of pollens in rice. Accumulation of DGDG has been reported in the pollen tube membranes of *Arabidopsis* (Botté et al. 2011) and in lily pollen during tube elongation (Nakamura et al. 2009). Similarly, high proportion of galactolipid (almost 50%) was also observed in pollen coat of two lines of *Brassica napus* (Evans et al. 1990). Furthermore, inhibition of galactolipid biosynthesis in *Arabidopsis* by an inhibitor (galvestine-1), which inhibits MGDG synthase, resulted in impaired pollen tube growth in vitro (Botté et al. 2011). In *Arabidopsis*, the N-terminal sequence of the DGD1(NDGD1) was reported to be required for the transfer of galactolipid between the chloroplast envelopes, thereby mediating the accumulation of DGDG in the thylakoid membrane (Dörmann et al. 1999; Kelly et al. 2016). On the other hand, the loss of DGD2 was not found to result in any impairment in the thylakoid membrane biogenesis (Kelly et al. 2003), suggesting the possible role of DGD2 in supplying DGDG to lipid compartment outside plastids. These results indicate that DGD2, without the N-terminal end, might be involved in supply of DGDG to extra-plastidic membranes and OsDGD2β could be particularly involved in synthesis of DGDG crucial for anther development in rice.

### Future prospects: hybrid rice breeding using *osdgd2β*

Heterosis breeding has been a powerful way of increasing productivity, enhancing nutrition and quality, and providing resistance to environmental stresses, thereby meeting the global food-security demands. Heterosis in rice can be achieved by using a cytoplasmic male sterility (CMS) line-based three-line system or a photoperiod/thermosensitive genic male sterile (PTGMS) line-based two-line system (Chen and Liu 2014; Huang et al. 2014; Bai et al. 2018). Despite tremendous success, both the systems have their own intrinsic drawbacks. CMS system is limited by narrow germplasm resources of restorer lines, difficulty to breed new traits into the parental lines, and poor genetic diversity between the CMS lines and restorer lines (Huang et al. 2014; Wang and Deng 2018). Whereas, two-line system is limited by vulnerability to fluctuating environmental conditions affecting production of hybrid seeds as well as PTGMS seeds (Chen and Liu 2014; Wang and Deng 2018). Another new system of rice breeding has recently emerged which uses the nuclear male sterility for hybrid seed production. This system overcomes the limitations posed by other system as it is temperature independent and has the ability to propagate male sterile seeds on a production scale (Chang et al. 2016a). Basically, it uses the recessive nuclear male-sterile lines, which can be engineered to form the female parent for hybrid seed production (Kim and Zhang 2018; Wang and Deng 2018). *osnp1*, a male sterile mutant, was transformed with the complementary functional gene (*OsNP1*) coupled with a gene to deactivate the transgenic pollen and a marker for identification of transgenic seeds (Chang et al. 2016a). This system has been shown to overcome the intrinsic drawbacks of both CMS and PTGMS systems, thereby showing great practical potentiality for hybrid rice breeding and production. In the present study, we demonstrated that *osdgd2β* plants are male sterile, hence could also be used in hybrid rice production as *osnp1*. In practice, any rice variety could be used for targeted gene editing using the CRISPR/Cas9 system to produce male sterile plants as shown in this study and further engineered for large scale production of male sterile seeds as shown in Additional file 8: Figure S7. Briefly, male sterile plants (*osdgd2β/−*) are generated by targeted mutagenesis of *OsDGD2β*, then crossed with its wild-type to produce transgene-free heterozygous fertile plants (*OsDGD2β/−*), which are self-crossed to produce homozygous male sterile seeds and plants (via genotyping of the *osdgd2β* mutation). Calli are induced from *osdgd2β* plant tissues (e.g. immature inflorescent) and transformed with a fertility restoring vector (pC_DR). The pC-DR consists of *OsDGD2β* (driven by its indigenous promoter) to complement the mutated gene, and a red fluorescence protein gene (*RFP,* driven by a seed, especially an aleurone, specific promoter (Kalla et al. 1994; Xu et al. 2016) to mark the transgenic seeds. The resulting transgenic, hemizygous plants (*osdgd2β/−;* pC-DR*) are fertile and could be crossed back to the male sterile plants (*osdgd2β/−*) to produce transgenic and non-transgenic seeds. The transgenic seeds are identified by the red fluorescence and used as a male sterility maintainer line for male sterile seed multiplication, while the transgene-free male sterile seeds are used commercially for hybrid seed production by crossing with a male parent (Additional file 8: Figure S7).

## Conclusion

In this report, we identified 5 genes encoding DGDG synthase in rice and analyzed their gene structure, motifs and domains in proteins. *OsDGD2β,* an anther specific gene, was found essential for pollen development and loss of function of this gene lead to male sterility in rice. This result confirms the importance of galactolipids in tissues other than photosynthetic membranes of higher plants. The male sterility conferred by loss of function of OsDGD2β could have huge potential application in breeding of rice using nuclear male sterility system.

## Methods

### In silico analysis: identification and analysis of DGDG synthase genes in rice

Identification of DGDG synthase genes in rice was conducted by searches in multiple databases such as Gramene (http://gramene.org/), KEGG (Kyoto Encyclopedia of genes and genomes) (http://www.genome.jp/kegg/kegg2.html), Plant metabolic network (https://www.plantcyc.org). The protein sequences of the *Arabidopsis* DGDG synthase genes (*AtDGD1* and *AtDGD2*) obtained from the TAIR database (https://www.arabidopsis.org/ are used as queries to BLAST against the rice genome in NCBI (National Center for Biotechnology Information’s GenBank) (http://www.ncbi.nlm.nih.gov/) and Gramene database to confirm the genes and check for any non-redundant genes. The information on genes such as their position, protein length, and transcript number were investigated on Gramene database and the protein sequences were used to analyse the molecular weight and isoelectric point in ExPASy database (https://web.expasy.org/compute_pi/). The gene structure (exon-intron distribution) was studied on The Gene Structure Display Server (GSDS_2.0_) (http://gsds.cbi.pku.edu.cn). EBI's HMMER tool (https://www.ebi.ac.uk/Tools/hmmer/) was used for domain analysis to identify and locate the Pfam domain region using the protein sequences. The multiple sequence alignment was performed using MEGA 7.0 with CLUSTALW and maximum likelihood (ML) under the Jones-Taylor-Thornton (JTT) model was used to create the phylogenetic tree. The conserved protein motifs of DGDG proteins were analysed using the MEME 4.12 with number of different motifs as 15 and default parameters of minimum motif width as 6 and a maximum motif width set to 50. The tissue specific expression data of the DGDG synthase genes in rice, *Arabidopsis* and maize was obtained from RGAP (Rice genome annotation project), TAIR (The *Arabidopsis* Information Resource), and maizeGDB (maize genetics and genomics database) database, respectively. The transcriptomic genetic expression of genes encoding DGDG synthase at four different stages of anther development in rice was obtained from (Deveshwar et al. 2011).

### Laboratory experiment

#### CRISPR based mutagenesis of *OsDGD2β*

CRISPR Vector pHUN4c12 (Xu et al. 2014) was used to construct genome editing vectors for the targeted mutagenesis of *OsDGD2β*. The genome specific sequence of sgRNA (D2b-T/−B) were designed using CRISPR-P 1.0 (http://crispr.hzau.edu.cn/CRISPR/) targeting the third exon of *OsDGD2β* and annealing buffer 5X (Beyotime, Nantong, China) was used to anneal the oligos together. BsaI-HF (NEB) was used to digest the vector pHUN4c12 and was ligated to the annealed oligos using the T4 DNA ligase. The ligated vector, hence named pHUN4c12s:OsDGD2β, was then transformed into the chemically competent DH5α and the insertion of sgRNA oligos was verified by sequencing using primer (seq-F). The recombinant vector was then introduced into the chemically competent *Agrobacterium* (EHA105) and transformed into rice callus induced from a *japonica* rice genotype Xidao #1 (Liu et al. 2019) using *Agrobacterium* mediated transformation.

#### Identification and growth of mutants

CTAB method was used to extract the DNA from leaf. PCR primer pair D2b-F/ -R was used for amplification and sequencing of the targeted fragment to study the type of mutation. The target fragment of the bi-allelic mutant was cloned into the pGEM®-T Easy vector, transformed into *E. coli* and sequenced to identify the mutations. The truncation in the protein translation in the mutants were analysed using ExPASy translate tool (http://web.expasy.org/translate/). The T_0_ mutant plants were cropped after flowering and the ratoons were separated from the stalk and grown for subsequent studies.

#### Characterization of mutant phenotype

The spikelets were collected on the day of heading in tubes containing the fixative solution FAA (Formalin/Acetic acid/Alcohol) and stored at room temperature. The structures of flower and anther were studied under a simple microscope. The pollen fertility was studied by staining with 1% IKI (iodine-potassium-iodide) solution and observed under a compound microscope. The female fertility in the mutants was investigated by emasculating some of the panicles on the day of flowering and cross pollinating with pollens from the wild-type.

For TEM, anthers were pre-fixed by immersing in 2.5% glutaraldehyde in Phosphate-buffered saline (PBS, 0.1 M pH 7.0) for at least 4 h. 1% osmium tetraoxide was used to post-fixation for 2 h and washed with PBS. The anthers were dehydrated first with gradient ethanol, then with absolute acetone, and finally with spur resin. Semi-thin sections (2 μm) were obtained using ultra-microtome with a glass knife, stained with 0.5% toluidine blue and observed under a microscope. Ultra-thin sections (100 nm) were obtained using the LEICA EM UC7 ultratome, stained by uranyl acetate and alkaline lead citrate for 5–10 min, respectively, and observed under transmission electron microscope (Hitachi H-7650, Tokyo, Japan).

#### Characterization of mutant phenotype

Flag leaves were collected on the day of flowering and chlorophyll content was quantified according to (Lichtenthaler and Wellburn 1983) using 96% (v/v) ethanol. The photosynthetic rate, stomatal conductance, and transpiration rate was analysed using the portable photosynthetic system (LiCOR LI-6400XT, Lincoln, USA) according to manufacturer’s instructions.

#### Lipid composition analysis

Total lipid extraction was performed according to Wang and Benning 2011, taking 30 mg of anther or leaf. This crude lipid was either directly converted to FAMEs or first separated by TLC using 80 mL developing solvent composed of acetone, toluene, water (91 mL: 30 mL: 7.5 mL) and the DGDG spot, visualized by iodine staining, was then scraped out and converted to FAME. 1 mL 1% MeOH/H_2_SO_4_ was added to each sample capped tightly and kept in a water-bath (80 °C) for 1 h. 0.9% NaCl and 1 mL hexane was added to each sample, centrifuged, and the FAMEs dissolved in upper hexane layer was analysed using GC-FID according to (Li et al. 2013).

#### Gene expression analysis

Leaf and anther samples collected on the day of flowering were used to extract total RNA using RNAprep pure kit (Tiangen Biotech, Beijing, China). Reverse-transcription of RNA was performed with Primescript RT reagent kit (Cat. # RR0037). Lightcycler (Roche Illuminator, Penzberg, Germany) was used to perform qRT-PCR using Hieff™ qPCR SYBR® master mix (Yeasen, China). The experiment was conducted with three biological replications, each with three technical replicates. *OsActin* was used as internal reference and the relative expression levels were measured using the 2^-ΔΔCt^ analysis method. The primer sequences are included in the Additional file 9: Table S9.

#### Protein subcellular localization of OsDGD2β

cDNA of *OsDGD2β* was PCR amplified (Primer: D2b-cD-F/−R) using KOD Neo plus polymerase (TOYOBO) and initially inserted into pMD18-T vector for sequence confirmation. Primer pair D2b-HR-F/−R was used for PCR amplification of the cDNA (with sequence overlapped) from the recombinant vector and inserted into pGFP-EGFP via XhoI and NcoI restriction sites using the homologous recombination method (Vazyme Biotech co., Ltd), resulting in an C-terminal fusion with GFP, i.e. 35S:OsDGD2β:GFP. The protoplast was extracted from 10 days old rice seedlings and the construct was transfected into the extracted protoplasts using polyethylene glycol (He et al. 2016), incubated for 14 h and observed under a confocal scanning microscope (LSM780, Zeiss, Germany).

## Additional files

Additional file 1: **Table S8.** Predicted sequences features of OsDGDG proteins and annotations in various database. Accessed on 05/02/2019. (XLS 67 kb)

Additional file 2: **Figure S1.** In silico analysis of DGDG synthase genes and proteins in *Arabidopsis* and rice. A Phylogenetic relationship between DGDG proteins. B Intron/exon organization of DGDG synthase genes, Introns and exons are represented by black and green coloured boxes, respectively, UTR are coloured blue. C distribution of conserved motifs and domain of DGDG protein. Each motif is represented in coloured box represented by a number. Length of box does not correspond to motif length and order of motifs corresponds to position of motifs in individual protein sequence. Glycos_transf_1 is the only domain present in both rice and *Arabidopsis* represented in purple colour. (PDF 353 kb)

Additional file 3: **Figure S2.** Tissue specific expression in rice (A), *Arabidopsis* (B), and maize (C). Relative expression values for rice, *Arabidopsis* and maize were obtained from RGAP, TAIR and maizeGDB database. D shows the expression of *OsDGD2β* and *OsDGD1β* at PMA (pre-meiotic anther), MA (meiotic anther), SCP (anther with single celled pollen) and TPA (anther with tri-nucleate pollen) stages of anther development in rice. Microarray expression values were obtained from Deveshwar et al. (2011). (PDF 63 kb)

Additional file 4: **Figure S3.** Thin Layer Chromatography (TLC) of total lipid extracted from anther in wild-type cultivar Xidao #1 (1–3) and its mutant *osdgd2β-1* (4–6)*.* The protocol for extraction and identification for lipid spots [using reference figure (Ref)] was adopted from Wang and Benning (2012). Black arrow head shows the flow direction of chromatography. MGDG, Monogalactosyldiacylglycerol; DGDG, Digalactosyldiacylglycerol; SQDG, Sulfoquinovosyldiacylglycerol; PI, Phosphatidylinositol; PE, Phosphatidylethanolamine; PC, Phosphatidylcholine. (PDF 1939 kb)

Additional file 5: **Figure S4.** Measurement of photosynthetic parameters in a wild-type cultivar Xidao #1 and its mutants *osdgd2β-1* and *osdgd2β-2*. All values represent means ± standard deviations. (PDF 34 kb)

Additional file 6: **Figure S5.** Measurement of chlorophyll content in leaf of a wild-type cultivar Xidao #1 and its mutants *osdgd2β-1* and *osdgd2β-2.* All values represent means ± standard deviations. (PDF 32 kb)

Additional file 7: **Figure S6.** Panicles on the day of flowering in a wild-type cultivar Xidao #1 (A) and its mutant *osdgd2β-1* (B). C shows seed-set on emasculated panicle of mutant 15 days after cross pollinated with wild-type pollen. (PDF 7420 kb)

Additional file 8: **Figure S7.** Schematic diagram showing breeding of hybrid rice using nuclear male sterility system. WT-A & WT-B could be any female or male line, respectively, of a hybrid variety. pH-D is a CRISPR/Cas9 gene editing vector (*pHUN4c12:OsDGD2β*) used for targeted mutagenesis of *OsDGD2β,* and pC-DR is a fertility restoring vector consisting of *OsDGD2β* to complement the mutated gene, and a red fluorescence protein gene (*RFP*) for seed sorting. The transgenic seeds with pC-DR could be sorted by the red fluorescence. The male sterile, transgene-free seeds could be used in hybrid seed production by crossing with WT-B. Blue shaded box represents transgenic lines. + and – represents wild-type and mutated *OsDGD2β*, respectively. ⨂ represents self-pollination. Detailed information is provided in the article (Discussion 3.3). (PDF 37 kb)

Additional file 9: **Table S9.** List of oligos and primers (XLS 49 kb)

## Data Availability

All data supporting the conclusions of this article are provided within the article and its Additional file 1. The gene expression data for rice, Arabidopsis and maize are available in http://rice.plantbiology.msu.edu; https://www.arabidopsis.org; https://maizegdb.org, respectively.

## Abbreviations

Cas9: CRISPR associated protein 9
CMS: Cytoplasmic male sterility
CRISPR: Clustered regularly interspaced short palindromic repeats
DGDG: Digalactosyldiacylglycerol
GC-FID: Gas chromatography flame ionization detector
GFP: Green fluorescence protein
MGDG: Monogalactosyldiacylglycerol
PCD: Programmed cell death
TEM: Transmission electron microscopy
TLC: Thin layer chromatography
