# Supplementary figures and images for: OsDGD2β is the Sole Digalactosyldiacylglycerol Synthase Gene Highly Expressed in Anther, and its Mutation Confers Male Sterility in Rice
Source: Rice (N Y). 2019 Aug 14;12:66. doi: 10.1186/s12284-019-0320-z (PMC6694320; doi:10.1186/s12284-019-0320-z)

**A**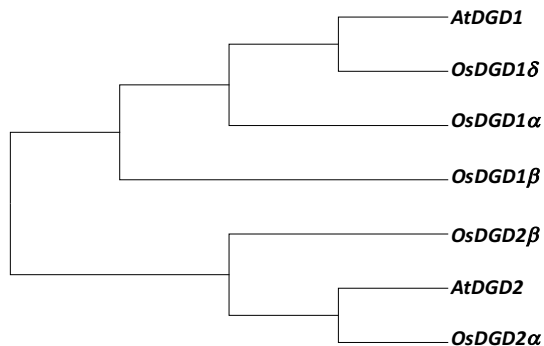**B**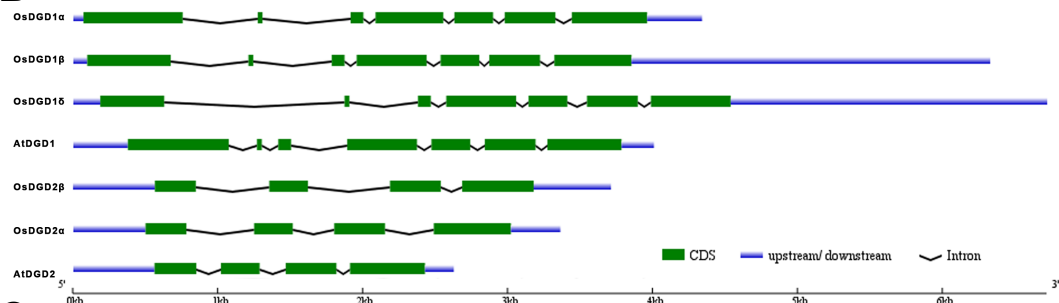**C****Proteins**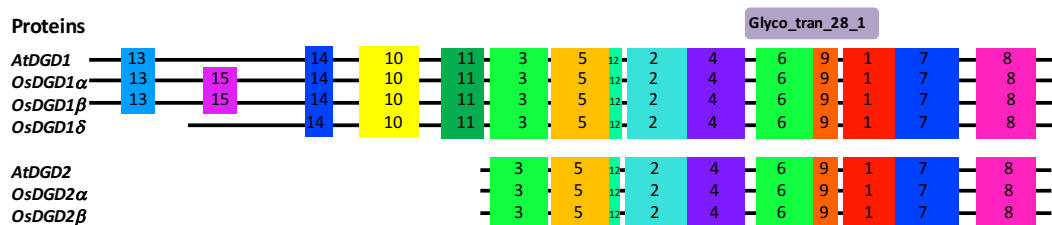

Supplement: Supplementary file 2 — Figure S1. In silico analysis of DGDG synthase genes and proteins in Arabidopsis and rice. A Phylogenetic relationship between DGDG proteins. B Intron/exon organization of DGDG synthase genes, Introns and exons are represented by black and green coloured boxes, respectively, UTR are coloured blue. C distribution of conserved motifs and domain of DGDG protein. Each motif is represented in coloured box represented by a number. Length of box does not correspond to motif length and order of motifs corresponds to position of motifs in individual protein sequence. Glycos_transf_1 is the only domain present in both rice and Arabidopsis represented in purple colour. (PDF 353 kb) [file 12284_2019_320_MOESM2_ESM.pdf]

**A**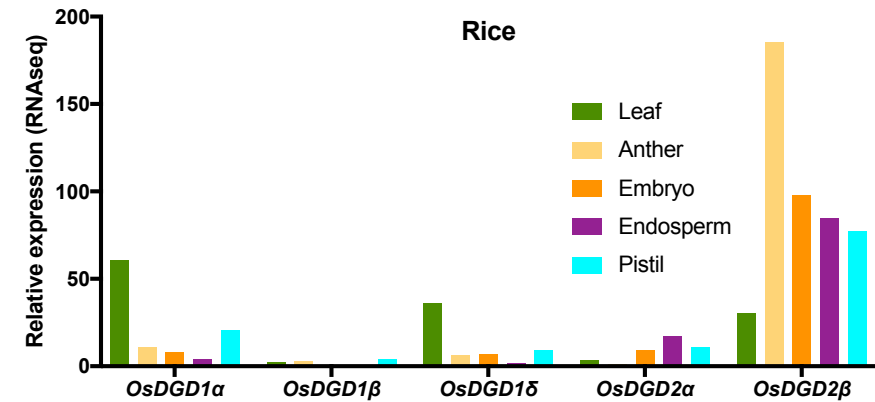**B**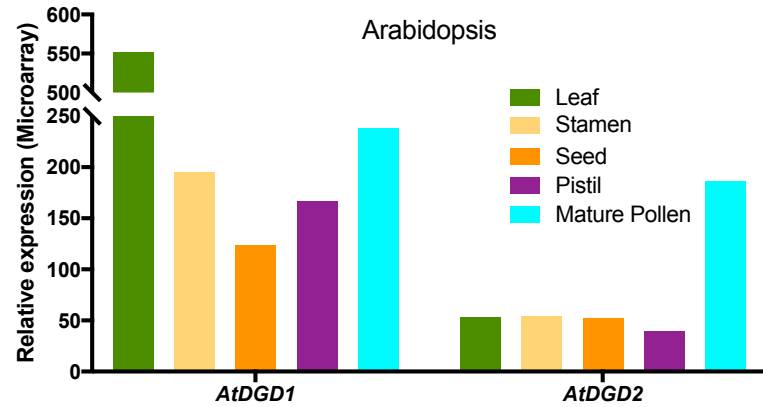**C**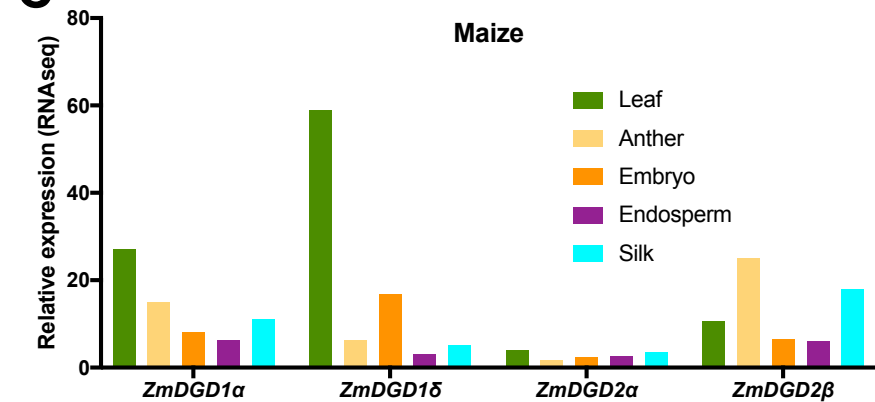**D**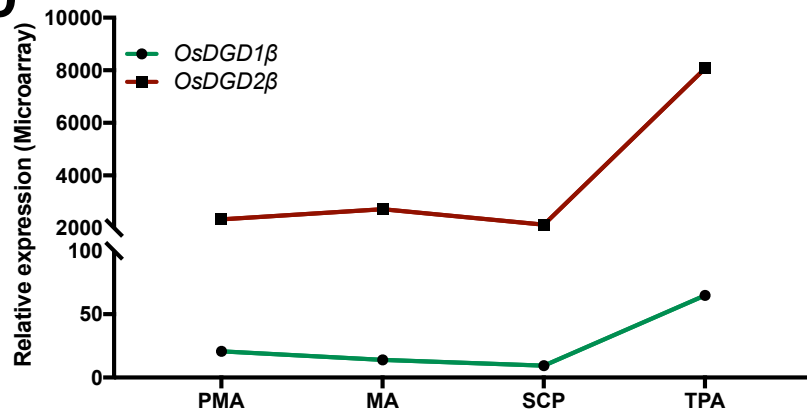

Supplement: Supplementary file 3 — Figure S2. Tissue specific expression in rice (A), Arabidopsis (B), and maize (C). Relative expression values for rice, Arabidopsis and maize were obtained from RGAP, TAIR and maizeGDB database. D shows the expression of OsDGD2β and OsDGD1β at PMA (pre-meiotic anther), MA (meiotic anther), SCP (anther with single celled pollen) and TPA (anther with tri-nucleate pollen) stages of anther development in rice. Microarray expression values were obtained from Deveshwar et al. (2011). (PDF 63 kb) [file 12284_2019_320_MOESM3_ESM.pdf]

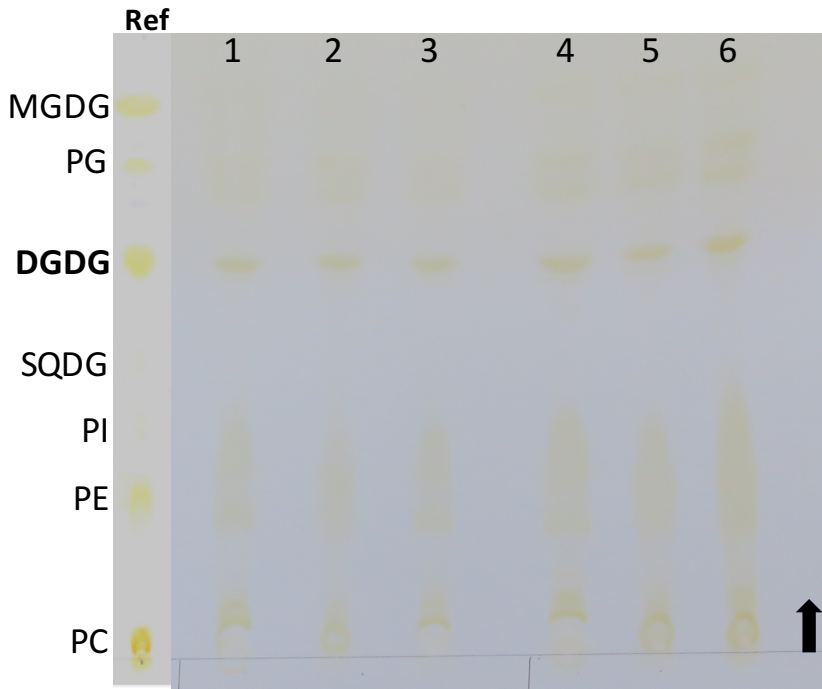

Supplement: Supplementary file 4 — Figure S3. Thin Layer Chromatography (TLC) of total lipid extracted from anther in wild-type cultivar Xidao #1 (1–3) and its mutant osdgd2β-1 (4–6). The protocol for extraction and identification for lipid spots [using reference figure (Ref)] was adopted from Wang and Benning (2012). Black arrow head shows the flow direction of chromatography. MGDG, Monogalactosyldiacylglycerol; DGDG, Digalactosyldiacylglycerol; SQDG, Sulfoquinovosyldiacylglycerol; PI, Phosphatidylinositol; PE, Phosphatidylethanolamine; PC, Phosphatidylcholine. (PDF 1939 kb) [file 12284_2019_320_MOESM4_ESM.pdf]

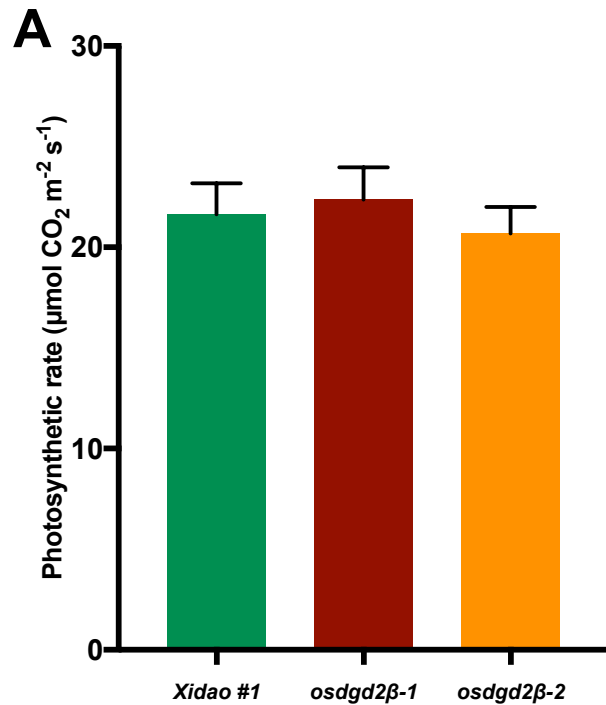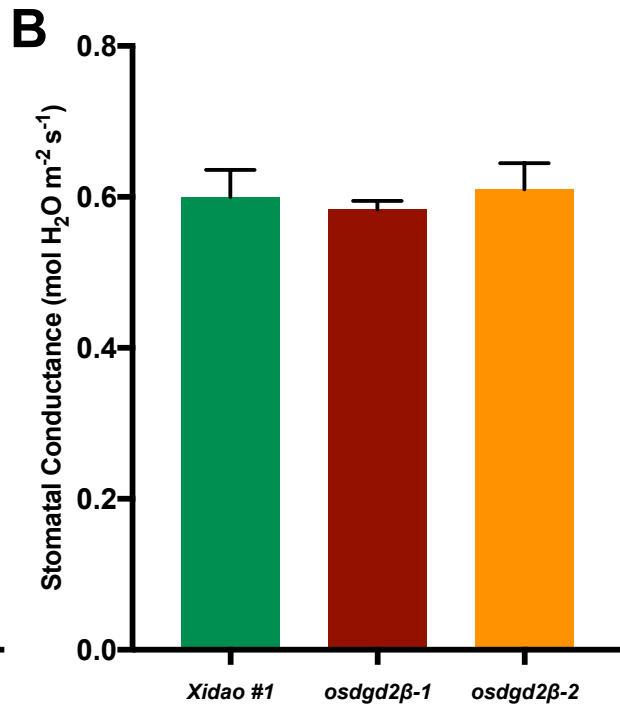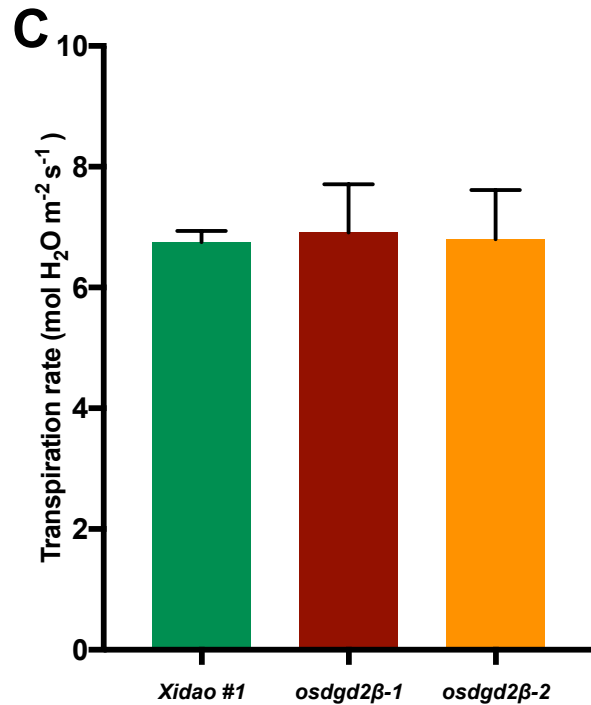

Supplement: Supplementary file 5 — Figure S4. Measurement of photosynthetic parameters in a wild-type cultivar Xidao #1 and its mutants osdgd2β-1 and osdgd2β-2. All values represent means ± standard deviations. (PDF 34 kb) [file 12284_2019_320_MOESM5_ESM.pdf]

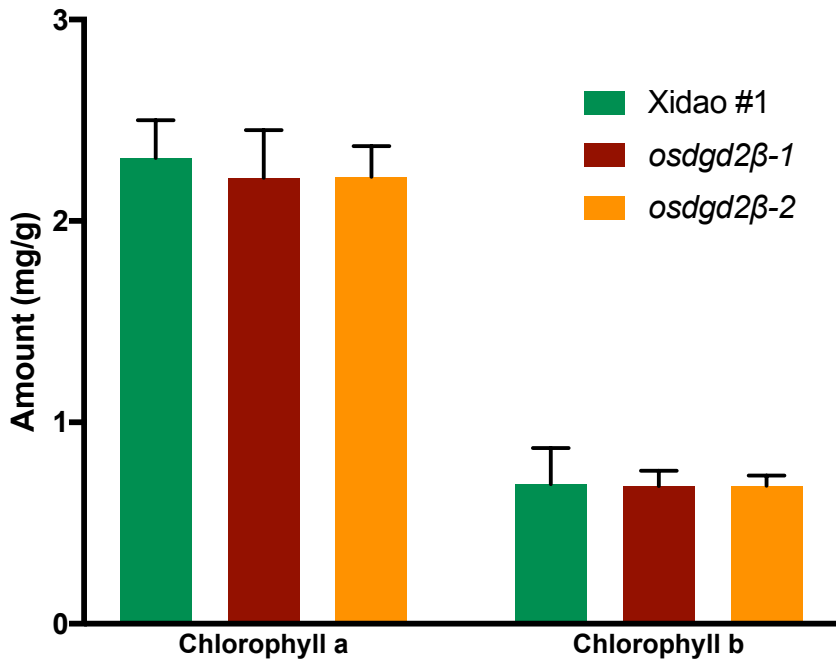

Supplement: Supplementary file 6 — Figure S5. Measurement of chlorophyll content in leaf of a wild-type cultivar Xidao #1 and its mutants osdgd2β-1 and osdgd2β-2. All values represent means ± standard deviations. (PDF 32 kb) [file 12284_2019_320_MOESM6_ESM.pdf]

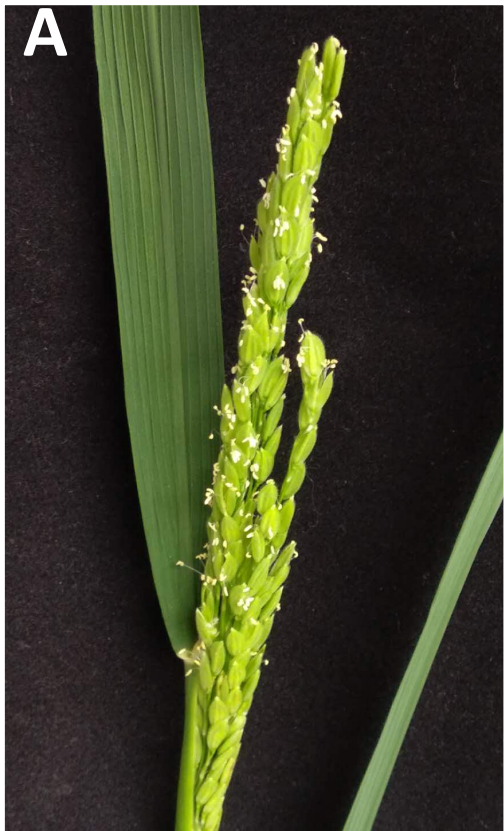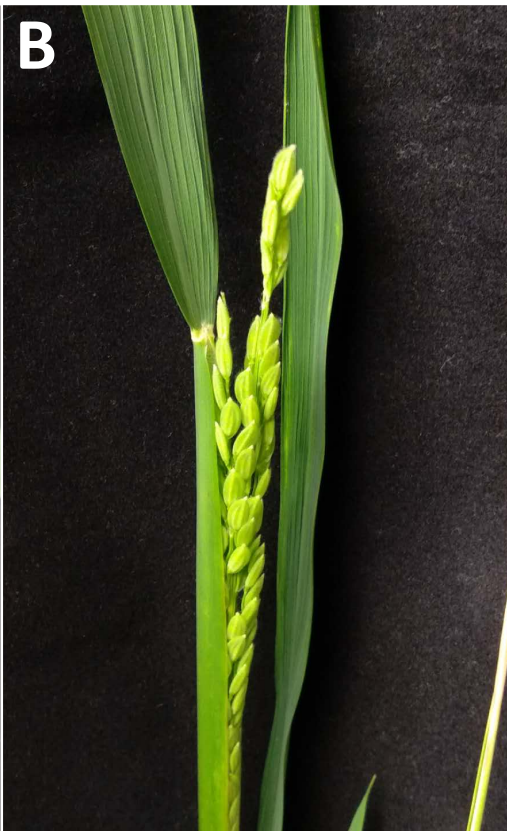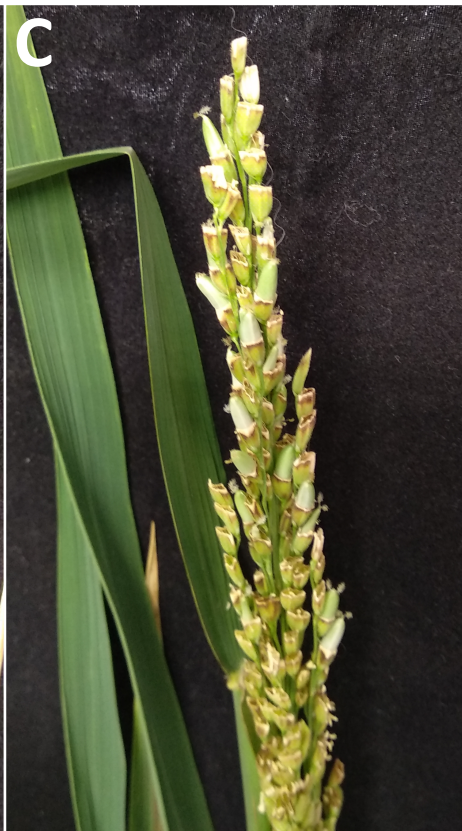

Supplement: Supplementary file 7 — Figure S6. Panicles on the day of flowering in a wild-type cultivar Xidao #1 (A) and its mutant osdgd2β-1 (B). C shows seed-set on emasculated panicle of mutant 15 days after cross pollinated with wild-type pollen. (PDF 7420 kb) [file 12284_2019_320_MOESM7_ESM.pdf]

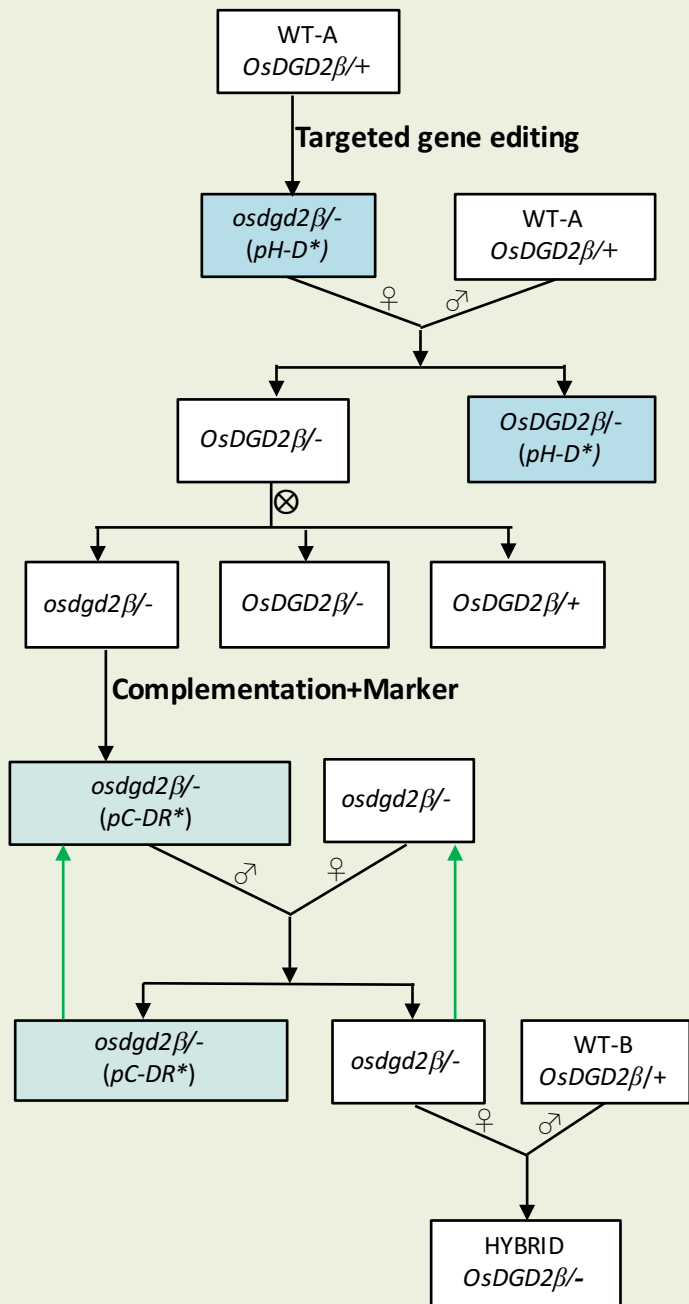

Supplement: Supplementary file 8 — Figure S7. Schematic diagram showing breeding of hybrid rice using nuclear male sterility system. WT-A & WT-B could be any female or male line, respectively, of a hybrid variety. pH-D is a CRISPR/Cas9 gene editing vector (pHUN4c12:OsDGD2β) used for targeted mutagenesis of OsDGD2β, and pC-DR is a fertility restoring vector consisting of OsDGD2β to complement the mutated gene, and a red fluorescence protein gene (RFP) for seed sorting. The transgenic seeds with pC-DR could be sorted by the red fluorescence. The male sterile, transgene-free seeds could be used in hybrid seed production by crossing with WT-B. Blue shaded box represents transgenic lines. + and – represents wild-type and mutated OsDGD2β, respectively. ⨂ represents self-pollination. Detailed information is provided in the article (Discussion 3.3). (PDF 37 kb) [file 12284_2019_320_MOESM8_ESM.pdf]
